# No good Markov strategies for Büchi objectives in countable MDPs

**DOI:** 10.1007/s10479-026-07226-6

**Published:** 2026-05-09

**Authors:** Stefan Kiefer, Richard Mayr, Mahsa Shirmohammadi, Patrick Totzke

**Affiliations:** 1 University of Oxford, Oxford, United Kingdom; 2 University of Edinburgh, Edinburgh, United Kingdom; 3 IRIF & CNRS, Paris, France; 4 University of Liverpool, Liverpool, United Kingdom

**Keywords:** Markov decision processes, Strategy complexity, Repeated reachability, Büchi

## Abstract

We study countably infinite Markov decision processes with Büchi objectives, which ask to visit a given subset of states infinitely often. A question left open by T.P. Hill (1979) is whether there always exist $\varepsilon$-optimal Markov strategies, i.e., strategies that base decisions only on the current state and on the clock (the number of steps taken so far). We provide a negative answer to this question by constructing a non-trivial counterexample.

## Introduction

***Background*** Markov decision processes (MDPs) are a standard model for dynamic systems that exhibit both stochastic and controlled behavior (Puterman, 1994). MDPs play a prominent role in numerous domains, including artificial intelligence and machine learning (Sigaud & Buffet, 2013; Sutton & Barto, 2018), control theory (Abbeel & Ng, 2004; Blondel & Tsitsiklis, 2000), operations research and finance (Bäuerle & Rieder, 2011; Katehakis et al., 2019; Schäl, 2002), and formal verification (Baier & Katoen, 2008; Clarke et al., 2018). In an MDP, the system starts in the initial state and makes a sequence of transitions between states. Depending on the type of the current state, either the controller gets to choose a distribution over successor states, or the next state is chosen randomly according to a defined distribution. By fixing a strategy for the controller, one obtains a probability space over infinite system executions, also called *runs* of the MDP. The goal of the controller is to optimize the expected value of some objective function on the runs.

The type of strategy needed for an optimal (resp. $\varepsilon$-optimal) strategy for some objective is called the *strategy complexity* of the objective. There are different types of strategies, depending on whether one can take the whole history of the run into account (history-dependent, H), or whether one is limited to a finite amount of memory (finite memory, F) or whether decisions are based only on the current state (memoryless, M). Moreover, we distinguish strategies based on whether the controller can randomize (R) or is limited to deterministic choices (D). The simplest type, MD, refers to memoryless deterministic strategies. *Markov strategies* are strategies that base their decisions only on the current state and the number of steps in the history of the run. Thus they do use infinite memory, but only in a very restricted form by maintaining an unbounded step-counter (also called *the clock*). For finite MDPs, there exist optimal MD-strategies for many (but not all) objectives (Chatterjee et al., 2004a, b; Chatterjee & Henzinger, 2012; Puterman, 1994), but the picture is more complex for countably infinite MDPs (Kiefer et al., 2017; Ornstein, 1969; Puterman, 1994).

Unless otherwise stated, we consider the *Büchi* objective, where one wants to visit a given subset *F* of the states infinitely often. In the simpler *Reachability* objective one just wants to reach a set *F* at least once.

***Previous work on 2-player stochastic games*** Our recent result in Kiefer et al. (2025) shows a very strong upper bound on the strategy complexity of Büchi objectives in concurrent (aka simultaneous-move) 2-player stochastic games with a countable number of states. For Maximizer, $\varepsilon$-optimal strategies for Büchi require just one bit of public memory if the game graph is acyclic, and only the clock plus one bit of public memory for general game graphs. These results assume that Minimizer’s action sets are finite, but Maximizer’s action sets may be infinite. Moreover, Maximizer’s strategy can be made deterministic if the game is turn-based (aka alternating-move). This deterministic clock-plus-one-bit upper bound trivially carries over to countably infinite MDPs, even if the action sets are infinite (Kiefer et al., 2025).

For concurrent stochastic games, corresponding *lower bounds* have long been known. In the “Bad Match” game (Maitra & Sudderth, 1996; Thuijsman, 1992), both Markov strategies and all strategies with finite (private) memory are worthless. I.e., these cannot guarantee any positive attainment for Maximizer, even though the initial state has value 1. See also (Kiefer et al. (2025), Sec. 3) for a detailed discussion. The “Bad Match” is a concurrent Büchi game with finitely many states and finite action sets. If one instead considers finite-state turn-based stochastic games with finite action sets then there always exist optimal MD Maximizer strategies for the Büchi objective (Chatterjee & Henzinger, 2012), and this trivially carries over to finite-state MDPs with finite action sets (see also (Puterman, 1994)).

***Previous work on MDPs*** The situation is more complex for MDPs with a countably infinite number of states and/or infinite action sets. For countably infinite MDPs, optimal strategies (where they exist) and $\varepsilon$-optimal strategies for Reachability can be chosen MD (Ornstein, 1969; Puterman, 1994). Similarly, optimal strategies for Büchi (where they exist) can be chosen MD (Kiefer et al., 2017). However, $\varepsilon$-optimal strategies for Büchi require infinite memory (cannot be chosen FR); cf. Kiefer et al. (2017); Krčál (2009).

**Fig. 1:**
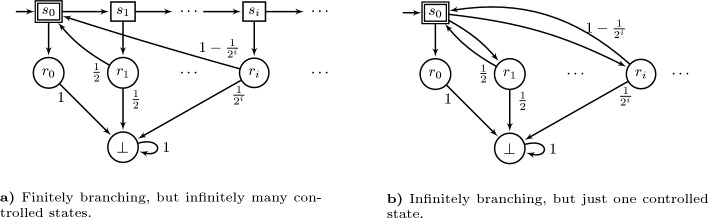
Two MDPs where $\varepsilon$-optimal strategies for Büchi require infinite memory. Let $F=\{s_0\}$ be the set of goal states. Here and throughout the paper we indicate goal states by double borders, and controlled states by rectangles

### Example 1

Consider the MDPs in Figure 1. Every finite-memory (FR) strategy will only attain probability 0 for Büchi in these examples (Kiefer et al., 2017). However, there exists an $\varepsilon$-optimal Markov strategy for every $\varepsilon >0$: At the *i*-th time that state $s_0$ is visited, pick the successor state $r_{i+k}$ where *k* is some sufficiently large number depending on $\varepsilon$, e.g., $k=\lceil \log _2(1/\varepsilon )\rceil$. For example b) this can easily be done with a step-counter since $s_0$ can only be visited for the *i*-th time in step $2(i-1)$. Similarly, for example a) under the strategy as above, state $s_0$ can only be visited for the *i*-th time in step $\sum _{j=1}^{i-1} (k+j+1)$.

**Fig. 2:**
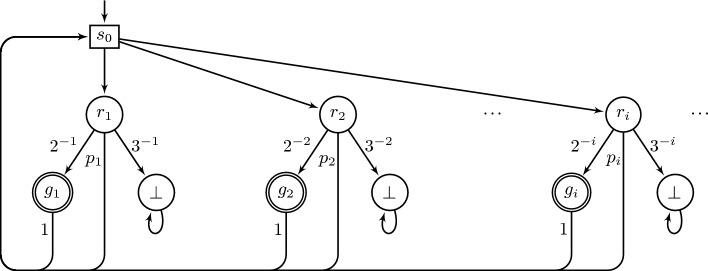
An MDP where $\varepsilon$-optimal strategies for Büchi require infinite memory. The transition probability $p_i$ stands for $1 - 2^{-i} - 3^{-i}$. The state $s_0$ is the only controlled state.

### Example 2

Consider the MDP from Figure 2, taken from (Hill 1999, Example 4.2). Every FR-strategy attains only probability 0 of Büchi. Moreover, the strategy that, in state $s_0$, subsequently picks $r_1, r_2, \ldots$ also attains probability 0, unlike in Example 1. But a different infinite-memory strategy achieves a positive probability. Indeed, let $\sigma$ be the strategy that, in $s_0$, picks $2^1$ times $r_1$ and then $2^2$ times $r_2$ and ...$2^i$ times $r_i$ etc. This strategy $\sigma$ achieves a positive probability of Büchi. (In more detail, $\sigma$ achieves a positive probability of never falling in a losing sink $\bot$, and in almost all of the remaining runs it visits a goal state infinitely often.) Note that $\sigma$ is a Markov strategy.

***The open problem*** While $\varepsilon$-optimal strategies for Büchi in the MDPs in Examples 1 and 2 require infinite memory, Markov strategies suffice here. Such examples led to the question whether there always exists a family of $\varepsilon$-optimal Markov strategies for Büchi in all countably infinite MDPs.

A partial answer was given by Hill (1979) (Proposition 5.1), who showed that $\varepsilon$-optimal Markov strategies for Büchi exist in the special case where the MDP contains only a *finite* number of controlled states. (However, the player can still have infinite action sets.) This result applies to the MDPs from Example 2 and Figure 1b), but not directly to the one in Figure 1a).

The question for general countable MDPs was stated as an open problem in (Hill 1979, p.158, l.4) and mentioned again in (Hill 1999, Q1 in Section 5).

***Our contribution*** We provide a negative answer to the open problem by presenting a non-trivial counterexample. We construct an MDP with a countably infinite number of states such that the step-counter (i.e., the clock) from the initial state $s_0$ is implicit in every state. In particular, this MDP is acyclic. Moreover, every state has an out-degree of at most 2. In other words, all action sets are of size $\le 2$ and all distributions over successor states have a finite support of size $\le 2$. While the initial state $s_0$ has value 1 wrt. Büchi, there are no $\varepsilon$-optimal Markov strategies from $s_0$ for any $\varepsilon <1$. I.e., Markov strategies are worthless here. In combination with the example from Figure 1, this shows that, for countable MDPs (even with finite action sets), neither finite memory (FR) nor Markov strategies are sufficient in general.

## Preliminaries

Let $\mathbb {R}$ and $\mathbb {N}$ denote the sets of real and natural numbers, respectively. A *probability distribution* over a countable set *S* is a function $f:S\rightarrow [0,1]$ with $\sum _{s\in S}f(s)=1$. We write $\mathcal {D}(S)$ for the set of all probability distributions over $S$.

For a set *S* we write $S^*$ (resp. $S^\omega$) for the set of all finite (resp. infinite) sequences of elements in *S*. We use slightly generalized regular expressions for sets of sequences, e.g., if $s_0 \in S$ we may write $s_0 S^\omega$ for the set of infinite sequences starting with $s_0$.

***Markov decision processes*** A *Markov decision process* (MDP) ${\mathcal {M}}=(S,S_\Box ,S_\ocircle ,{\longrightarrow },P)$ consists of a countable set $S$ of *states*, which is partitioned into a set $S_\Box$ of *controlled states* and a set $S_\ocircle$ of *random states*, a *transition relation* ${\longrightarrow }\subseteq S\times S$, and a *probability function* $P:S_\ocircle \rightarrow \mathcal {D}(S)$. We write $s{\longrightarrow }{}s'$ if $(s,s')\in {\longrightarrow }$, and refer to $s'$ as a *successor* of *s*. We assume that every state has at least one successor. The probability function *P* assigns to each random state $s\in S_\ocircle$ a probability distribution $P(s)$ over its (non-empty) set of successor states. A *sink in* ${\mathcal {M}}$ is a subset $T \subseteq S$ closed under the ${\longrightarrow }$ relation, that is, $s\in T$ and $s{\longrightarrow }s'$ implies that $s'\in T$.

An MDP is *acyclic* if the underlying directed graph $(S,{\longrightarrow })$ is acyclic, i.e., there is no directed cycle. It is *finitely branching* if every state has finitely many successors and *infinitely branching* otherwise. (Literature in gambling theory (e.g., Hill (1979, 1999)) present MDPs with action sets where every action yields a distribution over successor states. Instead our notation (more common in computer science literature) uses explicit random states. Thus our *finitely branching* condition implies both that the action sets are finite and that all distributions over successor states have only finite support. I.e., this is a strong restriction that makes our obtained lower bound even stronger.) An MDP without controlled states ($S_\Box =\emptyset$) is called a *Markov chain*.

***Strategies and Probability Measures*** A *run* $\rho$ is an infinite sequence $s_0s_1\cdots$ of states such that $s_i{\longrightarrow }{}s_{i+1}$ for all $i\in \mathbb {N}$; write $\rho (i){\mathop {=}\limits ^{\text {{{def}}}}}s_i$ for the *i*-th state along $\rho$. A *partial run* is a finite prefix of a run. We say that (partial) run $\rho$ *visits* $s$ if $s=\rho (i)$ for some *i*, and that $\rho$ starts in $s$ if $s=\rho (0)$.

A *strategy* is a function $\sigma :S^*S_\Box \rightarrow \mathcal {D}(S)$ that assigns to partial runs $\rho s\in S^*S_\Box$ a distribution over the successors $\{s'\in S\mid s{\longrightarrow }{} s'\}$. The set of all strategies in ${\mathcal {M}}$ is denoted by $\Sigma _{\mathcal {M}}$ (we omit the subscript and write $\Sigma$ if ${\mathcal {M}}$ is clear from the context). A (partial) run $s_0s_1\cdots$ is induced by strategy $\sigma$ if for all *i* either $s_i \in S_\Box$ and $\sigma (s_0s_1\cdots s_i)(s_{i+1})>0$, or $s_i \in S_\ocircle$ and $P(s_i)(s_{i+1})>0$.

An MDP ${\mathcal {M}}=(S,S_\Box ,S_\ocircle ,{\longrightarrow },P)$, an initial state $s_0\in S$, and a strategy $\sigma$ induce a probability space in which the outcomes are runs starting in $s_0$ and with measure ${\mathcal {P}}_{{\mathcal {M}},s_0,\sigma }$ defined as follows. It is first defined on *cylinders* $s_0 s_1 \ldots s_n S^\omega$, where $s_1, \ldots , s_n \in S$: if $s_0 s_1 \ldots s_n$ is not a partial run induced by $\sigma$ then ${\mathcal {P}}_{{\mathcal {M}},s_0,\sigma }(s_0 s_1 \ldots s_n S^\omega ) {\mathop {=}\limits ^{\text {{{def}}}}}0$. Otherwise, ${\mathcal {P}}_{{\mathcal {M}},s_0,\sigma }(s_0 s_1 \ldots s_n S^\omega ) {\mathop {=}\limits ^{\text {{{def}}}}}\prod _{i=0}^{n-1} \bar{\sigma }(s_0 s_1 \ldots s_i)(s_{i+1})$, where $\bar{\sigma }$ is the map that extends $\sigma$ by $\bar{\sigma }(w s) = P(s)$ for all $w s \in S^* S_\ocircle$. By Carathéodory’s theorem (Billingsley, 1995), this extends uniquely to a probability measure ${\mathcal {P}}_{{\mathcal {M}},s_0,\sigma }$ on the Borel $\sigma$-algebra $\mathcal {F}$ of subsets of $s_0 S^\omega$. Elements of $\mathcal {F}$, i.e., measurable sets of runs, are called *events* or *objectives* here. For $X\in \mathcal {F}$ we will write $\overline{X}{\mathop {=}\limits ^{\text {{{def}}}}}s_0S^\omega \setminus X\in \mathcal {F}$ for its complement and ${\mathcal {E}}_{{\mathcal {M}},s_0,\sigma }$ for the expectation wrt. ${\mathcal {P}}_{{\mathcal {M}},s_0,\sigma }$. We drop the indices wherever possible without introducing ambiguity.

***Strategy Classes*** Strategies are in general *randomized* (R) in the sense that they take values in $\mathcal {D}(S)$. A strategy $\sigma$ is *deterministic* (D) if $\sigma (\rho )$ is a Dirac distribution for all runs $\rho \in S^{*} S_\Box$. (Randomized vs. deterministic is also called mixed vs. pure in some literature.)

We formalize the amount of *memory* needed to implement strategies. Let $\textsf{M}$ be a countable set of memory modes, and let $\tau : \textsf{M}\times S\rightarrow \mathcal {D}(\textsf{M}\times S)$ be a function that meets the following two conditions: for all modes $\textsf{m}\in \textsf{M}$,

- for all controlled states $s\in S_\Box$, the distribution $\tau (\textsf{m},s)$ is over $\textsf{M}\times \{s'\mid s{\longrightarrow }{} s'\}$.
- for all random states $s\in S_\ocircle$, we have $\sum _{\textsf{m}'\in \textsf{M}} \tau (\textsf{m},s)(\textsf{m}',s')=P(s)(s')$.

The function $\tau$ together with an initial memory mode $\textsf{m}_0$ induce a strategy $\sigma _{\tau }:S^*S_\Box \rightarrow \mathcal {D}(S)$ as follows. Consider the Markov chain with the set $\textsf{M}\times S$ of states and the probability function $\tau$. A sequence $\rho =s_0 \cdots s_i$ corresponds to a set $H(\rho )=\{(\textsf{m}_0,s_0) \cdots (\textsf{m}_i,s_i) \mid \textsf{m}_0,\ldots , \textsf{m}_i\in \textsf{M}\}$ of runs in this Markov chain. Each $\rho s \in s_0 S^{*} S_\Box$ induces a probability distribution $\mu _{\rho s}\in \mathcal {D}(\textsf{M})$, the probability of being in state $(\textsf{m},s)$ conditioned on having taken some partial run from $H(\rho s)$. We define $\sigma _{\tau }$ such that $\sigma _{\tau }(\rho s)(s')=\sum _{\textsf{m},\textsf{m}'\in \textsf{M}} \mu _{\rho s}(\textsf{m}) \tau (\textsf{m},s)(\textsf{m}',s')$ for all $\rho s\in S^{*} S_\Box$ and all $s' \in S$.

We say that a strategy $\sigma$ can be *implemented* with memory $\textsf{M}$ if there exist $\textsf{m}_0 \in \textsf{M}$ and $\tau$ such that $\sigma _{\tau }=\sigma$. We define certain classes of strategies:

- A strategy $\sigma$ is *finite memory* (F) if there exists a finite memory $\textsf{M}$ implementing $\sigma$.
- A strategy $\sigma$ is *memoryless* (M) (also called *positional*) if it can be implemented with a memory of size 1. We may view M-strategies as functions $\sigma : S_\Box \rightarrow \mathcal {D}(S)$.
- A strategy $\sigma$ is *1-bit* if it can be implemented with a memory of size 2. Such a strategy is then determined by a function $\tau :\{0,1\}\times S\rightarrow \mathcal {D}(\{0,1\} \times S)$. Intuitively $\tau$ uses one bit of memory to capture two different modes.
- A strategy $\sigma$ is *Markov* if it can be implemented with the natural numbers $\mathbb {N}$ as the memory, and a function $\tau$ such that the distribution $\tau (\textsf{m},s)$ is over $\{\textsf{m}+1\}\times S$ for all $\textsf{m}\in \textsf{M}$ and $s\in S$. Intuitively, such a strategy depends only on the current state and the number of steps taken so far, i.e., it has access to a step-counter. We view Markov strategies as functions $\sigma : \mathbb {N} \times S_\Box \rightarrow \mathcal {D}(S)$. Note that such a strategy is generally not finite memory.
- A strategy $\sigma$ is *1-bit Markov* if it can be implemented with $\mathbb {N} \times \{0,1\}$ as the memory, and a function $\tau$ such that the distribution $\tau (n,b,s)$ is over $\{n+1\}\times \{0,1\}\times S$ for all $(n,b)\in \textsf{M}$ and $s\in S$. We view such strategies as functions $\sigma : \mathbb {N}\times \{0,1\} \times S_\Box \rightarrow \mathcal {D}(\{0,1\} \times S)$.

***Payoffs, Values, Optimality*** We are interested in strategies to maximize the expectation of a given measurable *payoff* function $f:S^\omega \rightarrow \mathbb {R}$, a random variable that assigns a real value to every run. The *value* of state $s$ (wrt. *f*) is the supremum of expected values of $f$ over all strategies:

$$ {\texttt{val}_{{\mathcal {M}},f}(s)} {\mathop {=}\limits ^{\text {{{def}}}}}\sup _{\sigma \in \Sigma }{\mathcal {E}}_{{\mathcal {M}},s,\sigma }(f), $$

For $\varepsilon \ge 0$ and $s\in S$, we say that a strategy $\sigma$ is $\varepsilon$*-optimal* iff ${\mathcal {E}}_{{\mathcal {M}},s,\sigma }(f) \ge {\texttt{val}_{{\mathcal {M}},f}(s)} -\varepsilon$ and *uniformly* $\varepsilon$-optimal iff this holds for every $s\in S$. A (uniformly) 0-optimal strategy is simply called (uniformly) *optimal*.

In this paper, we will need two types of payoff functions. The first is the *total reward*, a random variable given as $f(\rho ) {\mathop {=}\limits ^{\text {{{def}}}}}\sum _{t=0}^\infty \textit{r}(\rho (t))$, where $\textit{r}:S\rightarrow \mathbb {R}$ is some given *reward* function. A useful fact (Puterman (1994), Theorem 7.1.9) is that if $S$ is finite and the range of $\textit{r}$ is bounded then there exist optimal strategies (for total reward) which are memoryless and deterministic.

The second type of payoff functions we consider are those with range $\{0,1\}$. Each such payoff function $f$ uniquely identifies an objective (set of runs) ${\varphi }$ by viewing *f* as the characteristic function of ${\varphi }$, i.e., $f(\rho )=1$ if $\rho \in {\varphi }$ and 0 otherwise. Then ${\mathcal {E}}_{{\mathcal {M}},s,\sigma }(f) = {\mathcal {P}}_{{\mathcal {M}},s,\sigma }({\varphi })$. We call this the *probability of achieving* ${\varphi }$ (using strategy $\sigma$ starting from the state $s$) and simply write ${\texttt{val}_{{\mathcal {M}},{\varphi }}(s)} = {\texttt{val}_{{\mathcal {M}},f}(s)} = \sup _{\sigma \in \Sigma }{\mathcal {P}}_{{\mathcal {M}},s,\sigma }({\varphi })$.

Our main focus are *reachability* (sometimes also called *goal*) and *Büchi* objectives, which are determined by a set of states $F\subseteq S$ and defined as follows. Let us slightly abuse notation and identify *F* with its characteristic function, i.e., $F(s)=1$ if $s\in F$.

- The *reachability* objective is to visit *F* at least once during a run. The corresponding payoff is $f(\rho ){\mathop {=}\limits ^{\text {{{def}}}}}\max _{t\in \mathbb {N}}F(\rho (t))$, and we define $\texttt{Goal}(F) {\mathop {=}\limits ^{\text {{{def}}}}}\{\rho \in S^{\omega } \mid \max _{t\in \mathbb {N}}F(\rho (t))=1\}$;
- The *Büchi* objective is to visit *F* infinitely often. The corresponding payoff function is $f(\rho ){\mathop {=}\limits ^{\text {{{def}}}}}\limsup _{t\rightarrow \infty }F(\rho (t))$, and we let ${\texttt {B}\ddot{\texttt {u}}{\texttt {chi}}}(F){\mathop {=}\limits ^{\text {{{def}}}}}\{\rho \in S^{\omega }\mid \limsup _{t\rightarrow \infty } F(\rho (t))=1\}.$

## The lower bound

In this section we solve Hill’s problem ( (Hill, 1979) and (Hill (1999), Q1) by exhibiting an MDP where the initial state has value 1 wrt. the Büchi objective, but every Markov strategy achieves this objective with probability 0.

### Theorem 1

There is a finitely branching countable MDP ${\mathcal {M}}$ such that ${\texttt{val}_{{\texttt {B}\ddot{\texttt {u}}{\texttt {chi}}}(F)}(s_0)} = 1$, but for every Markov strategy $\sigma$, we have ${\mathcal {P}}_{{\mathcal {M}},s_0,\sigma }({\texttt {B}\ddot{\texttt {u}}{\texttt {chi}}}(F)) = 0$. I.e., Markov strategies are worthless for Büchi.

The MDP ${\mathcal {M}}$ from Theorem 1 is constructed such that in every state *s* the number of steps from the start state $s_0$ is implicit in *s*. Hence using a clock does not confer any advantage to a strategy starting in $s_0$, and thus Markov strategies are equally good as memoryless strategies. In particular, the MDP ${\mathcal {M}}$ is acyclic.

It follows from the acyclicity of ${\mathcal {M}}$ and results in Kiefer et al. (2025) that, for every $\varepsilon >0$, $\varepsilon$-optimal strategies for Büchi from $s_0$ in ${\mathcal {M}}$ can be chosen as deterministic 1-bit strategies. However, later in this section we also show how to construct such strategies explicitly for our MDP ${\mathcal {M}}$.

First we construct an acyclic MDP where $\varepsilon$-optimal memoryless strategies do not exist, and subsequently we generalize it to solve Hill’s problem, by modifying this MDP such that the step-counter from $s_0$ is implicit in the states.

### Intuition and outline of the proof

**Fig. 3:**
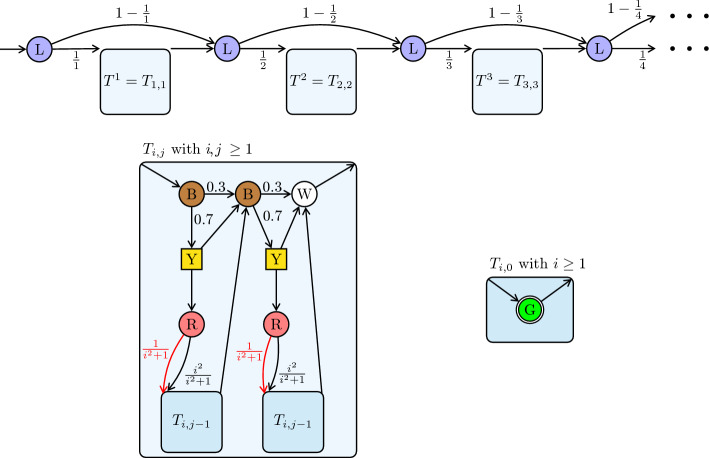
The MDP $\mathcal {M}$ consists of a chain alternating between blue states and trees $T^n$, for growing $n\in \mathbb {N}$. The trees $T^n$ are defined inductively in terms of subtrees $T_{i,j}$ with $i=n$ and $j\in \{0,1,\ldots ,n\}$.

We define a countably infinite MDP $\mathcal {M}$ as follows. The construction begins with a chain alternating between distinct blue states and height-*n* “trees” $T^n$, for growing $n \in \mathbb {N}$, as shown at the top of Figure 3. Blue states are labeled with *L*. The start state, $s_0$, of $\mathcal {M}$ is the leftmost (blue) state in this chain.

**Fig. 4:**
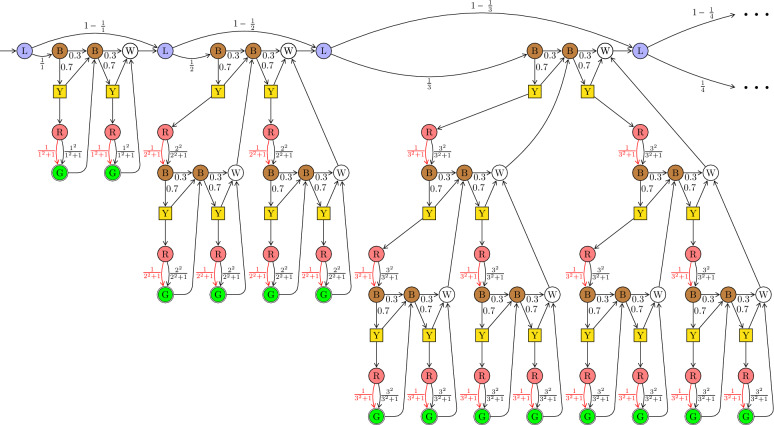
This acyclic MDP $\mathcal {M}$ is the unfolding of the inductive construction in Figure 3, showing explicitly the first three trees $T^1, T^2, T^3$. For $\mathcal {M}$ there are $\varepsilon$-optimal deterministic 1-bit strategies for the Büchi objective ${\texttt {B}\ddot{\texttt {u}}{\texttt {chi}}}(F)$ where *F* contains exactly all green states. No MR-strategy achieves even a positive probability.

Each tree $T^n = T_{n,n}$ is defined inductively based on subtrees $T_{n,j}$ with $j\in \{0,\ldots ,n\}$; see the bottom of Figure 3. For $i,j\ge 1$, the subtree $T_{i,j}$ is defined in terms of two copies of subtrees $T_{i,j-1}$, together with a collection of other states, again distinguished by colors: brown (B), yellow (Y), red (R), and white (W); see the bottom left of Figure 3. The probability 0.7 and its complementary value 0.3 have been fixed for the sake of concreteness; the proof could be adapted if 0.7 were replaced by any number in the interval $(\frac{1}{2},1)$. For the base case, the tree $T_{i,0}$ with $i\ge 1$ consists of a single green state (G); see the bottom right of Figure 3. Figure 4 depicts the initial segment of $\mathcal {M}$ with trees $T^1$, $T^2$ and $T^3$.

The controlled states are exactly the yellow states (Y). Each red state (R) in $T_{i,j}$ has two outgoing transitions: a black (right) transition and a red (left) transition, both leading to subtrees $T_{i,j-1}$. The goal set *F* consists of the green states (G) (in $T_{i,0}$).

We consider the strengthened Büchi objective that asks to see *F* infinitely often and moreover that *no red transition* is taken. This corresponds exactly to the normal Büchi objective if we redirect every red transition to an infinite (losing) chain of non-green states (not depicted in Figure 4). The purpose of the strengthened Büchi objective and the red transitions is to make our analysis of $\mathcal {M}$ less cumbersome.

We first argue that no MR-strategy achieves a positive probability of the (strengthened) Büchi objective. Then we show that the MDP $\mathcal {M}$ can be modified so that no Markov strategy achieves a positive probability.

***Intuition behind the construction of*** $\mathcal {M}$ The objective, say ${\varphi }$, of visiting infinitely many green states and no red transition creates tension between trying to visit green states and avoiding too many red states (as red states incur a risk of taking a red transition). In the proof we need to show that no memoryless strategy strikes a good balance between these competing goals. On the one end of the spectrum, an MR-strategy might always choose the upward transition in the yellow states (which are the only controlled states). But such a strategy never visits a green state and therefore clearly violates ${\varphi }$. On the other end of the spectrum lies the “greedy” MR-strategy, which always chooses the downward transition in the yellow states, in order to visit as many green states as possible. Indeed, under this strategy, let $u_n$ denote the probability that, starting in the top-left brown state of $T^n$, no green state is visited in $T^n$. By induction (see the formal proof below) one can show that there exists a number $u<1$ such that $u_n \le u$ holds for all *n*. Considering the probability of the transitions emanating from the blue states (at the top), the expected overall number of visited green states is at least $\sum _{n = 1}^\infty \frac{1}{n} (1-u_n) \ge (1-u) \sum _{n = 1}^\infty \frac{1}{n} = \infty$. It is not hard to strengthen this statement so that the greedy strategy almost surely visits infinitely many green states. So the greedy strategy satisfies one part of ${\varphi }$, but it does so at the expense of visiting many red states. Red states, though, are associated with a risk of taking a red transition, and it is shown in the formal proof below that the greedy strategy almost surely ends up taking at least one (and indeed infinitely many) red transition(s).

***Good deterministic 1-bit strategies*** The two competing goals discussed in the previous paragraph can be balanced using a deterministic 1-bit strategy, which we describe in the following. This strategy, $\sigma _1$, sets its bit to 0 whenever a blue state (at the top) is entered. While the bit is 0, in each tree $T^n$ it maximizes the probability of visiting a green state by choosing the downward transition in the yellow states, thus accepting a certain risk of taking a red transition. However, if and when a green state in $T^n$ is visited, the bit is set to 1, and for the remaining sojourn in $T^n$ the strategy $\sigma _1$ chooses the upward transitions in the yellow states, thus avoiding any risk of a red transition in the remainder of $T^n$. Although $\sigma _1$ appears to visit fewer green states than the aforementioned “greedy” MR-strategy, $\sigma _1$ still visits infinitely many green states almost surely. This is because for each tree $T^n$, the two strategies have the same probability of visiting at least one green state in $T^n$. The strategy $\sigma _1$ can be improved, for each $\varepsilon >0$, to achieve ${\varphi }$ with probability at least $1-\varepsilon$, by fixing the bit to 1 in the first *k* trees $T^1, \ldots , T^k$, for a *k* that depends on $\varepsilon$. Thus the first *k* trees are virtually skipped, eliminating the risk of taking any red transition there. In this way one can make the risk of taking a red transition arbitrarily small, while still visiting infinitely many green states with probability 1. Hence, for every $\varepsilon >0$, there exists such a deterministic 1-bit strategy $\sigma _1$ such that ${\mathcal {P}}_{{\mathcal {M}},s_0,\sigma _1}({\texttt {B}\ddot{\texttt {u}}{\texttt {chi}}}(F)) \ge 1-\varepsilon$, and in particular ${\texttt{val}_{{\texttt {B}\ddot{\texttt {u}}{\texttt {chi}}}(F)}(s_0)} = 1$.

***No good MR-strategies*** We need to show that not only are the extreme MR-strategies described above inadequate but that every MR-strategy achieves ${\varphi }$ with probability 0. To this end, for each tree $T^n$, define two probabilities:

- $t_n$ (for “total success”): the probability that, starting in the top-left brown state of $T^n$, at least one green state but no red transition is visited in $T^n$;
- $d_n$ (for “death”): the probability that, starting in the top-left brown state of $T^n$, a red transition is taken in $T^n$.

A very technical proof shows that $d_n \ge 0.008 \cdot t_n$ holds for all *n*, and this key inequality captures the inability of *any* MR-strategy to strike an adequate balance between the mentioned competing goals. Indeed, one can show that for an MR-strategy to have a positive probability of not visiting any red transition, the series $\sum _{n=1}^\infty \frac{1}{n} \cdot d_n$ needs to converge; but to have a positive probability of visiting infinitely many green states, the series $\sum _{n=1}^\infty \frac{1}{n} \cdot t_n$ needs to diverge (in both cases, the factor $\frac{1}{n}$ is the probability of visiting the top-left brown node of $T^n$). By the inequality above, this is impossible.

***No good Markov strategies*** For the proof of Theorem 1, we also need to show that all Markov strategies achieve probability 0. To this end, we modify the MDP $\mathcal {M}$ so that for each state, all paths from the initial state $s_0$ to *s* have the same length. I.e., that the step-counter from the initial state $s_0$ is implicit in any state *s*. This can be achieved by replacing some transitions in $\mathcal {M}$ by longer chains consisting of non-green states. This modification does not change the fact that MR-strategies achieve probability 0. But since in the new MDP each state can only be visited at a certain time, which is known a priori, a step-counter does not help. Hence all Markov strategies, like MR-strategies, achieve ${\varphi }$ with probability 0.

### The formal proof

We follow the proof sketch above and first argue that, in the MDP $\mathcal {M}$, no MR-strategy achieves a positive probability for the objective of visiting *F* infinitely often and taking no red transition. Indeed, given an MR-strategy and a tree, we define two probabilities:

- *s* (for “survival”): the probability that, starting in the top-left brown state, no red transition in the tree is visited;
- *t* (for “total success”): the probability that, starting in the top-left brown state, at least one green state but no red transition in the tree is visited.

Trivially, $t \le s$. A key lemma is the following.

#### Lemma 2

Write $p {\mathop {=}\limits ^{\text {{{def}}}}}0.7$. For every MR-strategy $\sigma$ and every $n \in \mathbb {N}$, the tree $T^n$ satisfies:

$$ s \ \le \ a^{q t n^2}\;, $$

where $$a = 1 - \frac{1}{n^2 + 1}$$ and $$q = \frac{1}{9} (1-p)$$.

#### Proof

Fix any MR-strategy $\sigma$ and any $n \in \mathbb {N}$. For each $k \in \{0, \ldots , n\}$, the tree $T^n$ has $2^{n-k}$ height-*k* subtrees, for which we can define *s*, *t* analogously. We claim: for all $k \in \{0, \ldots , n\}$ the probabilities *s*, *t* in every height-*k* subtree of $T^n$ satisfy

1 $$\begin{aligned} s \le a^{(q t + \frac{1}{2} q t^2) k^2}\;, \end{aligned}$$

where $a = 1 - \frac{1}{n^2 + 1}$ and $q = \frac{1}{9} (1-p)$. Note that the claim (for $k=n$) implies the lemma.

We prove the claim by induction on *k*. For the base case, $k=0$, note that each height-0 subtree of $T^n$ consists of only a single green state. Hence $s = t = 1$, so the claim holds for $k=0$. For the inductive step, let $k \in \{1, \ldots , n\}$ and consider a height-*k* subtree, say *T*, of $T^n$. Let $T_0, T_1$ be the left and the right subtree of *T*, respectively; they have height $k-1$. In the two (yellow) topmost controlled states in *T*, the MR-strategy $\sigma$ chooses probabilities to visit $T_0, T_1$, respectively. Taking into account the two brown random states at the top, the probabilities to visit $T_0, T_1$ are $p_0, p_1 \le p$, respectively. In $T_0, T_1$, the strategy $\sigma$ employs MR-strategies that achieve probabilities $s_0, t_0$ and $s_1, t_1$, respectively, where $s_i, t_i$ are defined in the obvious way for $T_i$. By the induction hypothesis we have

2 $$\begin{aligned} s_i \ \le \ a^{q t_i (1 + \frac{1}{2} t_i) (k-1)^2} \qquad \text {for } i \in \{0,1\}. \end{aligned}$$

By the structure of the MDP $\mathcal {M}$ we have:

3 $$\begin{aligned} s \ &= \ (1 - p_0 + p_0 a s_0) (1 - p_1 + p_1 a s_1) \end{aligned}$$

4 $$\begin{aligned} t \ &= \ p_0 a t_0 (1 - p_1 + p_1 a s_1) + p_1 a t_1 (1 - p_0 + p_0 a s_0) - p_0 a t_0 p_1 a t_1 \nonumber \\&\le \ p_0 a t_0 + p_1 a t_1 - p_0 a t_0 p_1 a t_1 \end{aligned}$$

By combining (2) and (3) we obtain:

5 $$\begin{aligned} s \ \le \ \prod _{i=0}^1 \left( 1 - p_i + p_i a^{1 + q t_i (1 + \frac{1}{2} t_i) (k^2-2k)}\right) \end{aligned}$$

On the other hand, from (4) we obtain:

6 $$\begin{aligned} \begin{aligned} q t + \frac{1}{2} q t^2 \ &\le \ q \left( p_0 t_0 + p_1 t_1 - p_0 a t_0 p_1 a t_1 \right) + \frac{1}{2} q \left( p_0 a t_0 + p_1 a t_1 \right) ^2 \\&\le \ p_0 q t_0 \left( 1 + \frac{1}{2} p_0 t_0 \right) + p_1 q t_1 \left( 1 + \frac{1}{2} p_1 t_1 \right) \end{aligned} \end{aligned}$$

Further, since $a>0$, we have:

7 $$\begin{aligned} \ln \frac{1}{a} \ \le \ \frac{1}{a} - 1 \ = \ \frac{n^2 + 1}{n^2} - 1 \ = \ \frac{1}{n^2} \ \le \ \frac{1}{k^2} \end{aligned}$$

Let $i \in \{0,1\}$. By (7) we have:

$$\begin{aligned} \left( \ln \frac{1}{a}\right) p_i q t_i \left( 1 + \frac{1}{2} p_i t_i \right) k^2 \ \le \ q \left( 1 + \frac{1}{2}\right) \ \le \ \frac{1}{9} \cdot \frac{3}{2} \ < \ \frac{1}{2} \end{aligned}$$

Hence, using a bound on the exponential function (Lemma 3 below), we obtain:

$$\begin{aligned} a^{p_i q t_i \left( 1 + \frac{1}{2} p_i t_i \right) k^2} \&= \ e^{- p_i (\ln \frac{1}{a}) q t_i \left( 1 + \frac{1}{2} p_i t_i \right) k^2} \\&\ge \ 1 - p_i + p_i e^{-(\ln \frac{1}{a}) q t_i \left( 1 + \frac{1}{2} p_i t_i \right) k^2 - (\ln \frac{1}{a})^2 q^2 t_i^2 \left( 1 + \frac{1}{2} p_i t_i \right) ^2 k^4} \\&\mathop {\ge }^\text {(7)} \ 1 - p_i + p_i e^{-(\ln \frac{1}{a}) q t_i \left( 1 + \frac{1}{2} p_i t_i \right) k^2 - (\ln \frac{1}{a}) \frac{9}{4} q^2 t_i^2 k^2} \\&= \ 1 - p_i + p_i a^{q t_i k^2 + q \left( \frac{1}{2} p_i + \frac{9}{4} q \right) t_i^2 k^2} \end{aligned}$$

By combining this inequality with (6) we obtain:

$$ a^{\left( q t + \frac{1}{2} q t^2\right) k^2} \ \ge \ \prod _{i=0}^1 \left( 1 - p_i + p_i a^{q t_i k^2 + q \left( \frac{1}{2} p_i + \frac{9}{4} q \right) t_i^2 k^2}\right) $$

Considering (5), we see that, in order to prove (1), it suffices to prove

$$\begin{aligned} 1 + q t_i \left( 1 + \frac{1}{2} t_i\right) (k^2-2k) \ &\ge \ q t_i k^2 + q \left( \frac{1}{2} p_i + \frac{9}{4} q \right) t_i^2 k^2 \qquad \text {for }i \in \{0,1\}. \end{aligned}$$

This inequality is equivalent to:

$$\begin{aligned} &  1 + q t_i k \left( \left( \frac{1}{2} - \frac{1}{2} p_i - \frac{9}{4} q \right) t_i k - 2 \left( 1 + \frac{1}{2} t_i \right) \right) \ &\ge \ 0 \\&\Longleftarrow \quad&1 + q t_i k \left( \left( \frac{1}{2} (1 - p) - \frac{9}{4} q \right) t_i k - 3 \right) \ &\ge \ 0 \\&\Longleftrightarrow \quad&1 + \frac{1}{9} (1-p) t_i k \left( \frac{1}{4} (1 - p) t_i k - 3 \right) \ &\ge \ 0 \\&\Longleftrightarrow \quad&\left( \frac{1}{6} (1-p) t_i k - 1 \right) ^2 \ &\ge \ 0 \end{aligned}$$

The left-hand side is a square, hence non-negative. This completes the induction proof. $\square$

The following elementary lemma from calculus was used in the proof of Lemma 2.

#### Lemma 3

For every $r \ge 0$ and $x \in [0, \frac{1}{2}]$ we have $e^{- r x} \ge 1 - r + r e^{-x -x^2}$.

#### Proof

Let $r \ge 0$ and $x \in [0, \frac{1}{2}]$. As $1+y \le e^y$ holds for all *y*, we have:

$$ 1 - r + r e^{-x -x^2} \ = \ 1 - r \left( 1 - e^{-x - x^2}\right) \ \le \ e^{-r \left( 1 - e^{-x-x^2}\right) } $$

Hence it suffices to prove that $x \le 1 - e^{-x - x^2}$, which is equivalent to $\ln (1-x) + x + x^2 \ge 0$. To prove the latter inequality, define $f(y) {\mathop {=}\limits ^{\text {{{def}}}}}\ln (1-y) + y + y^2$. Then we have $f(0) = 0$ and

$$ f'(y) \ = \ -\frac{1}{1-y} + 1 + 2 y \ = \ \frac{-1 + 1 - y + 2y - 2y^2}{1-y} \ = \ \frac{y (1-2 y)}{1-y} \ \ge \ 0 \ \text { for } y \in \left[ 0, \frac{1}{2}\right] . $$

By the fundamental theorem of calculus, it follows $f(x) = f(0) + \int _0^x f'(y) \; d y \ge 0$. $\square$

#### Lemma 4

Consider the acyclic MDP $\mathcal {M}$ shown in Figure 4. Let ${\varphi }$ be the objective of visiting infinitely many green states and no red transition.

1. For every MR-strategy $\sigma$, we have ${\mathcal {P}}_{{\mathcal {M}},s_0,\sigma }({\varphi }) = 0$.
2. ${\texttt{val}_{{\varphi }}(s_0)} = 1$ and for every $\varepsilon >0$ there exists a deterministic 1-bit strategy $\sigma _\varepsilon$ s.t. ${\mathcal {P}}_{{\mathcal {M}},s_0,\sigma _\varepsilon }({\varphi }) \ge 1-\varepsilon$.

#### Proof

First we prove item 1. Fix any MR-strategy $\sigma$. For each $n \in \mathbb {N}$, let $s_n, t_n$ denote the probabilities *s*, *t* for the tree $T^n$ under $\sigma$. Define also $d_n {\mathop {=}\limits ^{\text {{{def}}}}}1 - s_n$ (for “death”), which is the probability of taking at least one red transition starting in the top-left brown state of $T^n$. For the following estimate, observe that we have

8 $$\begin{aligned} \left( 1 - \frac{1}{x+1}\right) ^x \ = \ e^{x \ln \left( 1 - \frac{1}{x+1}\right) } \ \le \ e^{-\frac{x}{x+1}} \ \le \ e^{-\frac{1}{2}} \qquad \text {for } x \ge 1. \end{aligned}$$

By Lemma 2 we have for every *n*:

9 $$\begin{aligned} d_n \ = \ 1 - s_n \ \ge \ 1 - \left( 1 - \frac{1}{n^2 + 1} \right) ^{q t_n n^2} \ \mathop {\ge }^\text {(8)} \ 1 - e^{-\frac{1}{2} q t_n} \ \ge \ \frac{1}{4} q t_n \;, \end{aligned}$$

where the last inequality follows from the fact that $e^{-x} \le 1 - \frac{1}{2} x$ holds for $x \in [0, 1]$.

Denote by $G_n$ the indicator random variable such that

- $G_n = 1$ if the top-left brown state of $T^n$ is visited (coming from the previous blue state) and at least one green state in $T^n$ but no red transition in $T^n$ is visited;
- $$G_n = 0$$ otherwise.

Considering that the probability of visiting the top-left brown state of $T^n$ is $\frac{1}{n}$, we have ${{\mathcal {E}}} G_n = \frac{1}{n} \cdot t_n$, where ${{\mathcal {E}}}$ denotes expectation.

If $\sigma$ visits at least one red transition in $\mathcal {M}$ almost surely then the probability of ${\varphi }$ is 0. Therefore, suppose $\sigma$ achieves a positive probability, $\bar{r} > 0$, of visiting no red transition. Since $0 < \bar{r} = \prod _{n=1}^\infty \left( 1 - \frac{1}{n} \cdot d_n\right)$, the series $\sum _{n=1}^\infty \frac{1}{n} \cdot d_n$ converges. Thus:

$$\begin{aligned} {\mathcal {E}}\sum _{n=1}^\infty G_n \ = \ \sum _{n=1}^\infty {\mathcal {E}}G_n \ = \ \sum _{n=1}^\infty \frac{1}{n} \cdot t_n \ \mathop {\le }^\text {(9)} \ \frac{4}{q} \cdot \sum _{n=1}^\infty \frac{1}{n} \cdot d_n \ < \ \infty \end{aligned}$$

It follows that the probability that $\sum _{n=1}^\infty G_n$ diverges is 0. But on ${\varphi }$ the series $\sum _{n=1}^\infty G_n$ diverges. Hence the probability of ${\varphi }$ is 0. This completes the proof of item 1.

Towards item 2, we first define a suitable strategy, $\sigma$, that achieves a positive value (i.e., ${\mathcal {P}}_{{\mathcal {M}},s_0,\sigma }({\varphi })> 0$) and then improve it to obtain $\varepsilon$-optimal strategies $\sigma _\varepsilon$.

The strategy $\sigma$ acts independently in each tree $T^n$. In each tree $T^n$ the strategy $\sigma$ maximizes the probability of visiting exactly one green state. To this end, as long as $\sigma$ has not yet visited a green state in $T^n$, it chooses the downward transition emanating from the yellow controlled states; as soon as a green state in $T^n$ has been visited, $\sigma$ chooses the upward transition emanating from the yellow controlled states, thus avoiding any further visit of a green state or a red transition in $T^n$. This is a 1-bit strategy, as $\sigma$ remembers only whether a green state has already been visited in the current tree $T^n$. The bit is reset whenever a new tree is entered.

Next we show that $\sigma$ visits infinitely many green states with probability 1. Let $u_n$ denote the probability that, starting in the top-left brown state of $T^n$, no green state is visited in $T^n$. Define $u_0 = 0$. Since red or non-red transitions are unimportant for the current considerations, all height-*n* trees in $\mathcal {M}$ have the same structure, even when they are subtrees of different $T^m$. Therefore we have:

$$ u_n \ = \ (p u_{n-1} + 1 - p)^2 $$

Since the function $f(x) {\mathop {=}\limits ^{\text {{{def}}}}}(p x + 1 - p)^2$ is monotone on [0, 1], the sequence $(u_n)_n$ is non-decreasing and thus converges to the smaller fixed point, *u*, of *f*. Hence,

10 $$\begin{aligned} 0 \ \le \ u_n \ \le \ u \ = \ f(u) \ = \ \left( \frac{1-p}{p}\right) ^2 \ < \ 1 \qquad \text {for all }n \in \mathbb {N}\cup \{0\}. \end{aligned}$$

It follows that we have

11 $$\begin{aligned} \sum _{n=k}^\infty \frac{1}{n} (1-u_n) \ \ge \ (1-u) \sum _{n=k}^\infty \frac{1}{n} \ = \ \infty \qquad \text {for all }k \in \mathbb {N}. \end{aligned}$$

For every $k \in \mathbb {N}$, the probability that, starting in the blue state directly before $T^k$, no green state in $T^k, T^{k+1}, \ldots$ is visited is

$$ \prod _{n=k}^\infty \left( \frac{1}{n} \cdot u_n + \left( 1 - \frac{1}{n}\right) \right) \ = \ \prod _{n=k}^\infty \left( 1 - \frac{1}{n} (1-u_n) \right) \ \mathop {=}^\text {by (11)} \ 0\,. $$

It follows that $\sigma$ visits infinitely many green states with probability 1.

It now suffices to show that, with positive probability, $\sigma$ visits no red transition. Let $v_n$ denote the expectation, starting in the top-left brown state of $T^n$, of the number of red *states* (not red *transitions*) that are visited in $T^n$. Define $v_0 {\mathop {=}\limits ^{\text {{{def}}}}}0$. Since red or non-red transitions are unimportant for the current considerations, all height-*n* trees in $\mathcal {M}$ have the same structure, even when they are subtrees of different $T^m$. Therefore we have:

12 $$\begin{aligned} v_n \ &= \ p \left( 1 + v_{n-1} + u_{n-1} p (1 + v_{n-1}) \right) \ + \ (1-p) p (1 + v_{n-1}) \end{aligned}$$

We prove by induction that $v_n \le n$ holds for all $n \in \mathbb {N}\cup \{0\}$. The base case, $n=0$, holds by the definition of $v_0$. For the inductive step, let $n \ge 1$. We have:

$$\begin{aligned} v_n \ &\le \ p (n + u_{n-1} p n) + (1-p) p n &  \text {by (12) and the induction hypothesis}\\&\le \ p \left( n + \frac{(1-p)^2}{p} n\right) + (1-p) p n &  \text {by (12)} \\&= \ p n + (1-p)^2 n + p n - p^2 n \ = \ n \end{aligned}$$

Hence we have proved $v_n \le n$. It follows that the expectation, starting in the top-left brown state of $T^n$, of the number of red *transitions* visited in $T^n$ is at most $n \cdot \frac{1}{n^2 + 1}$. Thus the expected number of visited red transitions in the whole MDP $\mathcal {M}$ is at most $\sum _{n=1}^\infty \frac{1}{n} \cdot n \cdot \frac{1}{n^2 + 1} \le \frac{\pi ^2}{6}$. Hence there is $k \in \mathbb {N}$ such that the expected number of red transitions visited in $T^k, T^{k+1}, \ldots$ is less than 1. It follows from the Markov inequality that the probability to visit at least one red transition in $T^k, T^{k+1}, \ldots$ is less than 1. Hence the probability to visit at least one red transition in $\mathcal {M}$ is less than 1.

The strategy $\sigma$ from above can be improved to obtain an $\varepsilon$-optimal strategy $\sigma _\varepsilon$ for Büchi from $s_0$, i.e., ${\mathcal {P}}_{{\mathcal {M}},s_0,\sigma _\varepsilon }({\varphi }) \ge 1-\varepsilon$. We obtain $\sigma _\varepsilon$ by modifying the described strategy $\sigma$ such that, in the first *k* trees for some $k \in \mathbb {N}$, the upward transitions emanating from the yellow states are taken. By choosing a large but finite *k*, the risk of taking a red transition can be made arbitrarily small, while the probability of visiting infinitely many green states remains 1. $\square$

Finally, we are ready to prove our main result, Theorem 1.

#### Proof of Theorem 1

We describe how to modify the MDP $\mathcal {M}$ from Lemma 4 to obtain an MDP $\mathcal {M}_2$ with the claimed properties. First, eliminate the red transitions in $\mathcal {M}$ and change the objective to the normal Büchi objective. This can be done by redirecting all red transitions to an infinite (losing) chain of non-green states. Denote the resulting MDP by $\mathcal {M}_1$. For a state *s*, define its *depth* *d*(*s*) as the length of the *longest* path from the start state $s_0$ to *s*. In $\mathcal {M}_1$, each state has finite depth (this property does not follow from acyclicity alone). Now obtain $\mathcal {M}_2$ from $\mathcal {M}_1$ by replacing every transition that leads from a state $s_1$ to a state $s_2$ with $d(s_1) + 1 < d(s_2)$ by a chain (of non-green states) of length $d(s_2) - d(s_1)$. In this way, in $\mathcal {M}_2$, for every state *s*, all paths from $s_0$ to *s* have the same length *d*(*s*). Thus, instrumenting $\mathcal {M}_2$ with a step-counter would lead to an MDP isomorphic to $\mathcal {M}_2$. It follows that every Markov strategy for $\mathcal {M}_2$ could be replaced by an MR-strategy that achieves ${\texttt {B}\ddot{\texttt {u}}{\texttt {chi}}}(F)$ with the same probability. Observe that an MR-strategy for $\mathcal {M}_2$ directly translates to an MR-strategy for $\mathcal {M}$ that achieves the same probability. Hence, item 1 follows, as the existence of a Markov-, and hence MR-strategy that achieves positive probability would contradict Lemma 4.

Item 2 is shown by modifying the strategies $\sigma _\varepsilon$ from item 2 of Lemma 4 in the natural way.

#### Remark 1

The lower bound in our Theorem 1 requires an *infinite-state* MDP with action sets of size two. It does not apply to finite-state MDPs or even finite-state turn-based (aka alternating-move) 2-player stochastic games where memoryless Maximizer strategies suffice for the Büchi objective. For finite-state concurrent (aka simultaneous move) 2-player stochastic games, the “Bad Match” game of Thuijsman (1992); Maitra and Sudderth (1996); Kiefer et al. (2025) shows that Markov strategies do not suffice there either (though 1-bit Markov strategies do suffice (Kiefer et al. (2025), Sec. 3). Unlike in our result for MDPs, the lower bound for the “Bad Match” crucially requires a strategic opponent Minimizer player, whose response strategy depends on Maximizer’s strategy; cf. Kiefer et al. (2025, Sec. 3).

While an extra (public) memory bit (in addition to the clock) makes strategies qualitatively different, this does not imply fundamental obstacles from a computational point of view, e.g., for machine learning of strategies. This is because one could encode a *public* memory bit via having two copies of each state, one for each value of the bit. For computational problems, the main obstacle is rather the fact that the state space was countably infinite to begin with.
